# Case of Hepatic Affection

**Published:** 1810-03

**Authors:** John Crawford


### *Case of Hepatic Affection.* By Dr. Crawford *of Baltimore, America.*

[ Communicated to Dr. Bradley by Dr. R. J. Thornton. ]

MR. P. aged above 50, short, fat, a large head, and rather florid, came to this city from Norfolk, in the beginning of October, 1807; he lodged at the Indian Queen. I was sent for to visit him on the 13th, at 4 P. M. He was suffering from excessive pain in the hepatic region, extending to the parts in its vicinity, attended with nausea, and he had been some time without an alvine evacuation. His pulse was small and quick; ordered a saline purgative, which operated copiously, and afforded him sensible relief.

At noon, finding the febrile action still continue, notwithstanding the freedom with which the bowels were emptied,

℞. Camphoræ ℈j. spt. nitri dulcis ʒj. m. cochl. parv: pocillulo aquæ tepidæ edulcoratæ quaque hora sumat. This created no disturbance in the stomach, and in the evening the fever considerably remitted; at 10 o'clock P. M. he was free from uneasiness.

14th. He had a good night, but awoke this morning with a return of the uneasy sensations in the abdomen.

℞. Calomel. camphoræ aa ℈j. Pulv. antimon. ℈ss. Pulv. rhei ʒss. m. fiat pil. 16. æquales. Cujus capiat duas 2nda quaque hora donec alvus responderet mitte spt. nitri dulcis ʒj. cochl. parv. quaque hora sumat.

At noon he had taken eight of the pills, all of which were retained; the pain in the epigastrium distressing, and the pulse was quick and small, repr. mist. salin. ut heri addendo tinct. opii compos. ʒss.

He continued all the afternoon to increase in suffering from the pain in the epigastrium, and began to despond of life, having a constant *jactatio*: obtained only three small inefficient stools, although the mixture was quietly retained in the stomach. At 7 P. M. Dr. Littlejohn was called, and proposed venesection, from which, perhaps, I had been too much deterred by the smallness of the pulse. Eight ounces were taken from the arm. It was of a dark modena red, as it flowed from the orifice. The pulse soon after rose considerably. We gave him immediately afterwards a double dose of the saline mixture; but this was rejected. We had then recourse to ol. ricini, which remained, and in a short time operated freely. At 10 P. M. we we found him much easier, the paroxysm having gradually diminished and terminated by a gentle diaphoresis.

15th. We visited him at 7 A. M. He suffered much in the night from affliction in his abdomen; had since last evening several small stools without relief. We repeated the ol. ricini; by noon it had operated, but effected no benefit.

℞. Cal. ʒss. pulvs. opii gr. 3. m. f. l. a. pil. 6 æqs. unam 2nda quaque hora. S. Ordered an emollient enema, and warm salt in a bag to be applied to the part affected. At 4 P. M. some evacuations had been procured; but no diminution of suffering. The two first pills gave no disturbance. The third became so offensive he could not retain it. We agreed to give him the following purgative.

℞. Pulvs. jalapii. calomel. aa ℈ss. Sacchari ʒss. m.

This proved acceptable, and operated sufficiently, assisted by another enema. About five, the paroxysm which had endured from an early hour yesterday morning, twenty-four hours, with scarcely any abatement, now remitted, and he fell into a profound sleep. In this he continued until nearly 10 P. M. but alas! he again awoke, having a return of suffering; his pulse was 108, and no way diminished in strength, although he had been copiously purged. We endeavoured to alleviate his pain by diaphoretic anodynes, which, about 11 P. M. procured him a comfortable sleep, which continued until morning; but he awoke to a renewal of torment.

16th. At 6 A. M. the paroxysm recommenced. We repeated the saline mixture with the neutral salt; but this was not retained; we then gave him the same with cold infus. flor. cham. with no better effect. Ordered the pulv. jal. and cal. as before, which operated well; at 7 P. M. the pain was excessive; repeated the enema, and ordered an anodyne embrocation with volatile alkali.

17th. Had a restless night; a slight remission towards morning. Repeated the saline mixture; this occasioned flatulence; administered it with acid. mur. dilut. loco acid. limonum, which proved less offensive, and applied the anodyne embrocation diligently; pulse always soft, and ranged from 104 to 108; evening, having had no stool, repeated the enema.

18th. Again a bad night. The paroxysm had recurred with additional vehemence, and the pulse felt fuller than before; we ventured to take off about six ounces of blood, but an immediate failure of strength admonished us to desist. He continued very weak for several hours. When

 the the reaction was restored, his eyes evidenced an approaching delirium. At 1 P. M. he was completely deranged, and incapable of articulation; we applied epispastics to his ancles and the nape of his neck.

19th. Had a very restless night; this morning more composed; the epispastics operated effectually; having more evidence of apyrexia, we ordered a decoction of cinchona, which he took without inconvenience. At 1 P. M. he was able to pronounce a few words; we applied epispastics to his arms. In the afternoon he coughed with some energy, and brought up some matter that was very offensive in smell, and resembled pus. We gave him the decoct. cinchonæ with wine freely. At 7 P. M. he was nearly restored to speech, and complained of a more acute pain to the right of the serobiculus cordis than he had yet experienced.

20th. Had a tolerable quiet night, and was this morning perfectly sensible, and in the full possession of his speech; he desponded of recovery, and suffered much from the pain in the right epigastrium, but felt no inconvenience from lying on one side more than the other, nor was there any affection of either shoulder, such as accompanies an adhesion of the peritoneal coat of the liver to the intercostal muscles, or the parietes abdominis. The blisters on the extremities were dry, but that on the neck discharged freely. His pulse was weak, soft, and quick, and the debility was great. We continued the decoct. cinchonæ with wine, both of which proved very acceptable, and he used them unsparingly.

Evening, no abatement of any symptom, and a slight subsultus tendinum was perceiveable. The disease from this time until the twenty-third, proceeded with no other variation than is usual in the progress of such affections. He continued the decoction with wine; his bowels were kept open by enemas, and small portions of rhubarb were occasionally added to the doses of the decoction, with the desired effect. At 1 P. M. of this day, he had a smart rigor, which was succeeded by a higher degree of pyrexia than he had yet experienced. In the evening he was again deranged, and had two copious stools, by which he was much sunk. Whilst the pyrexia was advancing to its height, there was a strong pulsation in the epigastrium; that at the wrist was weak, and had an oscillation, which usually marks the formation of pus.

Being now satisfied that an abscess was forming, if not already formed in the superior gibbous part of the large lobe lobe of the liver, it became a question whether an opening was not immediately adviseable. We were pressed to undertake it by the patient, as affording the only possible means of saving his life, but were deterred from it by the following considerations.

The seat of pain was not confined to a particular spot, but occupied an area of, perhaps, more than an inch in diameter. There was no pain as before-mentioned, in either shoulder, and from these circumstances, no proof could be obtained that an adhesion existed between the liver and the external parts of the abdomen, which must be cut through to come at the abscess. Suppose these parts divided, and issue afterwards given to the contents of the abscess, wherein would the matter be received? The canula of a long trochar might, it is true, convey the principal part of it out of the body; but assuredly no small share would be admitted into the adjacent cavity. How far this would have defeated our purpose of remedying the disease, it is impossible to determine, but it certainly appeared to be too hazardous an undertaking. I have already stated, in my communication to you, on the seats and causes of disease, that in the Demerary hospital, I had often found an abscess in the internal parenchymatous part of the liver, where no adhesion to any of the surrounding parts was to be discovered. This strikingly appeared to be of the same description, and seemed to deny every hope to my mind, of the case being within the reach of human aid, we therefore declined the operation.

24th. The fever abated towards morning, when he obtained some quiet rest for several hours. About noon the paroxysm re-commenced, and continued increasing with great suffering through the whole day. Calomel camphr, and nitre in powders were administered, and quietly retained. This paroxysm was not ushered in with a rigor as yesterday; but 25th, was called to him this morning at four, when it recurred with great violence, to which a corresponding pyrexia supervened; a mercurial purgative was given, to open the bowels; an enema became requisite to effect this purpose. The fever abated towards evening; from its commencement the pain was very acute, still in the same situation. The pulse corresponded with the other symptoms. The fæces, of which he had two or three evacuations, were of a dark colour, and very offensive; this suggested a full dose of oleum ricini.

26th. No rest again until morning; the febrile action was less vigourous the whole of this day; the oil given last night did not operate; gave him a dose of calomel and jalap, but this was rejected. Appeased the irritation of the stomach by small and repeated doses of a solution sal. marin. in gruel, which, as I have often experienced, more effectually answered the purpose than perhaps any other means that could have been adopted; had no stool the whole of this day; towards evening he sensibly revived, the pulse was tranquil, and he felt stronger than since the commencement of the disease; this was a more marked intermission of suffering than had hitherto been noticeable; although a little attention to the foregoing account will evince that the malady, from its commencement, assumed, more or less, of a tertian type.

27th. Passed the night with little disquietude. The cathartics he took yesterday began to operate early this morning; either from this, or from some unusual exertion of the offending cause, he was purged smartly, and in consequence the pulse was much reduced; gave him preparations of the cinchona freely. Towards evening the perturbation of the system was renewed; and the matter expectorated, which had never ceased to be in large quantity, was tinged with blood; the cough was pertinacious all the evening.

28th. Some rest as usual towards morning, but the morbid distress was this day increasingly severe; the expectoration was more abundant than before, and still tinctured with blood.

29th. The febrile action was very little diminished the whole of this day; the matter expectorated, increased in quantity, was thinner, and instead of a sanguineous, was of a brown appearance. He complained less of pain, but there was a more circumscribed spot, of which, on pressure, he complained more than before. This was some inches anterior to the costæ. We had now a more marked proof of adhesion than had hitherto solicited our attention, but still insufficient, in our opinion, to warrant an operation, especially in so advanced a stage of the disease.

30th. Some sleep as usual towards morning, but also as usual, he awoke to renewed suffering. The sputation was again deeply coloured with florid blood, and in such quantity as to evidence great depredation in the seat of disease. In the evening, debility much increased, and his pulse became very feeble.

From this time to the period of his death, nothing unusual occurred, except that on the 3rd of November, he expectorated, with the pus, a substance of a fleshy appearance pearance as large as an almond. He expired on the sixth, the collection of pus, apparently in the thorax, and his incapacity to evacuate it, seeming to effect strangulation, and thus terminate his existence.

I sincerely lament that we were not allowed to examine the body after death; such diseases seldom occur here. To have ascertained whether any adhesion had been formed, so as to have made an incision safe, must have been an important acquisition. That an adhesion existed, when the seat of pain was at the advanced period of the disease more circumscribed, became sufficiently manifest, and that it was so situated, at the anterior part of the hepatic region, and clear of even the cartilages of the costæ, must have rendered an operation there perfectly safe. But repeated opportunities, both in the East and West Indies, of seeing issue given to the matter, when thus circumstanced, and unacquainted with any successful termination, I was deterred, at such a state of the malady, from countenancing the measure.

I was afterwards informed by very respectable medical connections of the patient, that the disease is not unfrequent at Norfolk, Virginia, and is there termed "Hepatic pleurisy." The appellation is certainly characteristic of the symptoms that occurred in this disease, but these do not fully correspond with what authors describe concerning it.

The symptoms adduced by Bianchi, in his Historia Hepatica, vol. I. p. 235, are "an acute fever with a pulse rather hard, difficult breathing; bloody sputation; a deep seated pain in the inferior part of the thorax, on the right; a very florid colour of the right, much more than of the left cheek. An icterical appearance of the whole surface, particularly of the eyes and tongue, and of all the secretions, especially of the urine, which is of a deep yellow colour. There is an intense dryness of the tongue and fauces; a very bitter taste in the mouth, and an insatiable thirst. Bile is constantly perspicuous in the sputation, and mixed with blood. An obtuse pain descends lower than the false ribs on the right side, a light pressure on which is complained of severely. Here a tumefaction is readily felt, occupying as much of the liver as in life is subjected to examination, which is hard and circumscribed. There is in that part an intense heat; a constant jactatio supervenes; and from the beginning, the thirst, the sense of heat, dryness of the fauces, and arridity on the whole surface of the body are importunate."

The circumstances wherein our case differs from the above, is, that the icterical appearances were not observable until an advanced stage of the disorder; the seat of it was much higher up; no tumefaction was discernible; there was no difference of colour between the right and left cheek; both were very florid during the paroxysms. The intermissions with us were of short duration it is true, but sufficiently marked, and are not noticed by our Author. The urine, as with him, was never of a deep yellow, but towards the close of a very red colour; in both, the lateritious sediment, which he mentions elsewhere, occurred, and was first discovered in our patient on the sixth day, although it might have happened earlier. It is certain that the blood in the sputation was not, in our case, discoverable until the fourteenth day. In every species of pleurisy, I have uniformly observed, that an early appearance of blood in sputation, before matter was formed, proved a favourable omen; but if it were deferred till pus was manifested, the greatest danger might be apprehended. This is conformable to the aphorism of Hippocrates, 340, as cited by De Gorter in his Medicina Hipocratica, p. 806. "A sanguinis sputo purissputum, malum." This remark has been made by numerous authors, and may be received as one of the best established medical facts. Towards the close of the disease, nearly all the symptoms enumerated by Bianchi were conspicuous.

I feel unspeakable concern that I did not, when first called, clearly comprehend the nature of the disease. Copious depletion, and an energetic use of mercury, particularly the ointment, might have, perhaps, more effectually prevented the fatal catastrophe. But an insulated case, the smallness and softness of the pulse, and the absence of these pathognomonic symptoms which decidedly characterise hepatitis, were strong obstacles to the adoption of what would have certainly been the most appropriate practice, as circumstances proved when it was too late. The truth of this will not admit of a question; but when we consider the absence of the symptoms, which would have proved a sure guide at the commencement of the disorder, when alone the treatment suggested could have been adviseable; that our patient was at a tavern, a stranger, and that the venesection we made did not accomplish much benefit; some consolation may be derived from these unpromising concomitants; indeed, as the early symptoms were so anomalous, it is little to be wondered that such measures measures were not suggested either to my mind, or that of my colleague.

I have been always of opinion, that a history of unsuccessful cases was more capable of enlightening the mind, and leading to a correction of errors, than of those which terminate favourably. They suggest more caution; they awaken more reflection, and they urge to a greater exertion of our own sagacity, and beget a disposition less to depend upon the "ipse dixit" of others. Histories of successful cases have too often, at least, as much of the honour of the writer in view, as of those which are unsuccessful, the advantage of the reader.

I am, &c.

JOHN CRAWFORD, M. D.

